# Colchicine vs pentoxifylline vs minocycline for generalized granuloma annulare: A retrospective cohort study

**DOI:** 10.1016/j.jdin.2023.12.002

**Published:** 2024-02-28

**Authors:** Jonathan D. Greenzaid, Matthew L. Hrin, Steven R. Feldman, Lindsay C. Strowd

**Affiliations:** a Department of Dermatology, Wake Forest University School of Medicine, Winston-Salem, North Carolina; b Department of Pathology, Wake Forest University School of Medicine, Winston-Salem, North Carolina; c Department of Social Sciences & Health Policy, Wake University Forest School of Medicine, Winston-Salem, North Carolina

**Keywords:** colchicine, drugs, general dermatology, granuloma annulare, immunomodulators, inflammation/inflammatory, medical dermatology, minocycline, pentoxifylline

*To the Editor:* Generalized granuloma annulare (GGA) is a variant of granuloma annulare (GA) that commonly occurs in children aged less than 10 years and adults aged above 40 years.^1^ GGA has more extensive lesions compared with GA, a chronic disease course recalcitrant to treatment, and limited treatment data.^1^^,^^2^ Although hydroxychloroquine, dapsone, methotrexate, and phototherapy have the most evidence in GGA, data regarding other systemic medications, such as colchicine, pentoxifylline, and minocycline, are generally limited to case reports and small case series (<10 patients).^2^, ^3^, ^4^, ^5^ We evaluated our experiences with colchicine, pentoxifylline, and minocycline in 42 patients with GGA.

Upon institutional review board approval, we retrospectively reviewed records of patients with biopsy-proven GGA treated with colchicine (GGA-C, *n* = 10), pentoxifylline (GGA-P, *n* = 22), and minocycline (GGA-M, *n* = 10) from 2010 to 2022 at our institution. Patients with localized disease were excluded. Outcomes were categorized as complete clearance (no active lesions), partial response (fewer and/or flattened lesions with reduced erythema), and no response. Mean duration to initial responses was compared among groups (GGA-C vs GGA-P vs GGA-M) using the Student *t* test, and response rates in the cohorts were compared using the χ^2^ analysis.

Demographic characteristics were similar between treatment groups (Table I). GGA presented as plaques (70% colchicine, 86% pentoxifylline, and 100% minocycline), with varying combinations of patches, papules, and nodules (Table I). Previous therapies among the cohorts included colchicine, topical corticosteroids, hydroxychloroquine, methotrexate, and dapsone (Table I).

**Table I:** Demographic and epidemiologic characteristics of 42 patients with GGA treated with colchicine, pentoxifylline, or minocycline

Characteristic	Value, *n* (%)		
	Colchicine	Pentoxifylline	Minocycline
Mean age at treatment, y (SD)	54 (8)	58 (14)	61 (7)
Sex			
Female	10 (100)	20 (91)	9 (90)
Male	0 (0)	2 (9)	1 (10)
Race			
Black	1 (10)	1 (5)	0 (0)
White	9 (90)	21 (95)	10 (100)
Mean disease duration, mo (SD)	97 (66)	81 (59)	85 (70)
Morphology			
Plaques	7 (70)	19 (86)	10 (100)
Papules	6 (60)	9 (41)	2 (20)
Patches	1 (10)	4 (18)	0 (0)
Nodules	1 (10)	1 (5)	0 (0)
Failed treatments before colchicine, pentoxifylline, or minocycline			
Topical corticosteroids	6 (60)	19 (86)	10 (100)
Hydroxychloroquine	4 (40)	3 (14)	1 (10)
Methotrexate	2 (20)	5 (23)	1 (10)
Dapsone	1 (10)	5 (23)	1 (10)

Patients were treated for a mean duration of 4.8 (GGA-C), 13.3 (GGA-P), and 6.4 months (GGA-M). Partial response to therapy was achieved in 50% (GGA-C), 27% (GGA-P), and 40% (GGA-M) of patients, and complete clearance was achieved in 10% (GGA-C), 5% (GGA-P), and 10% (GGA-M) of patients (Table II). Response rates were not significantly different between treatment groups (*P* = .33). Colchicine yielded faster initial responses (2.7 months) compared with minocycline (7.2 months, *P* = .013). Mean duration to initial response was similar when comparing pentoxifylline (4.2 months) versus colchicine (*P* = .22) and minocycline (*P* = .091). Gastrointestinal disturbance (20% GGA-C, 14% GGA-P, and 0% GGA-M) and dizziness (0% GGA-C, 5% GGA-P, and 0% GGA-M) were the only reported side effects (Table II). One colchicine responder received concomitant treatment with dapsone, and 3 pentoxifylline responders received concomitant therapy with either dapsone, methotrexate, or hydroxychloroquine. There was no difference in duration to initial response in responders receiving monotherapy versus therapy with multiple systemic medications (GGA-C: *P* = .55; GGA-P: *P* = .3). Six minocycline (60%) patients received concomitant ofloxacin and rifampin (ie, triple antibiotic therapy), and duration to initial response was comparable to minocycline monotherapy (*P* = .25).

**Table II:** Clinical response of patients with GGA treated with colchicine, pentoxifylline, or minocycline

Characteristic	Value, *n* (%)		
	Colchicine	Pentoxifylline	Minocycline
Patients treated with monotherapy	8 (80)	16 (73)	4 (40)
Patients achieving partial response	5 (50)	6 (27)	4 (40)
Mean duration of therapy to achieve partial response (mo)	2.7	7.2	4.2
Patients achieving complete clearance	1 (10)	1 (5)	1 (10)
Duration of therapy to achieve complete clearance (mo)	6	42	6
Patients reporting side effects	2 (20)	4 (18)	0 (0)

Although our study is limited by its sample size, lack of standardized outcomes at uniform time points, and limited racial diversity, this study bolsters the sparse pool of GGA treatment data. Furthermore, patients may not have been on therapy long enough to determine adequate response, as granulomatous diseases often require 6 to 12 months of therapy before achieving a response. In view of the relatively low complete clearance rates we report, further studies may consider evaluating the effectiveness of pentoxifylline, colchicine, and minocycline as adjunctive therapies in patients who partially respond to other therapies.

## Conflicts of interest

Steven R. Feldman has received research, speaking, and/or consulting support from Eli Lilly and Company, GlaxoSmithKline/Stiefel, AbbVie, Janssen, Alovtech, vTv Therapeutics, Bristol-Myers Squibb, Samsung, Pfizer, Boehringer Ingelheim, Amgen, Dermavant, Arcutis, Novartis, Novan, UCB, Helsinn, Sun Pharma, Almirall, Galderma, Leo Pharma, Mylan, Celgene, Ortho Dermatology, Menlo, Merck & Co, Qurient, Forte, Arena, Biocon, Accordant, Argenx, Sanofi, Regeneron, the National Biological Corporation, Caremark, Teladoc, BMS, Ono, Micreos, Eurofins, Informa, UpToDate, and the National Psoriasis Foundation. He is founder and part owner of Causa Research and holds stock in Sensal Health. Lindsay C. Strowd has received research, speaking, and/or consulting support from Pfizer, Novartis, Galderma, and Sanofi. The other authors have no conflicts to disclose.
